# Arthroscopic treatment successfully treats posterior elbow impingement in an athletic population

**DOI:** 10.1007/s00167-017-4563-1

**Published:** 2017-05-22

**Authors:** Jason L. Koh, Brad A. Zwahlen, David W. Altchek, Todd A. Zimmerman

**Affiliations:** 1 NorthShore Orthopaedic Institute, NorthShore University HealthSystem, 2650 Ridge Avenue Suite 2505, Evanston, IL 60201 USA; 2 California Sports and Orthopaedic Institute, 2999 Regent Street Suite 225, Berkeley, CA 94705 USA; 3 Department of Orthopaedic Surgery, Hospital for Special Surgery, 535 East 70th Street, New York, NY 10021 USA

**Keywords:** Elbow, Impingement, Arthroscopic, Debridement, Athletes

## Abstract

**Purpose:**

Posterior elbow impingement can cause disabling pain and limited motion during activities involving elbow extension. Less understood is whether arthroscopic treatment, compared to open surgery, can result in effective management of pain, loss of range of motion, and return athletes to previous levels of activity. This study determined whether arthroscopic debridement is a safe and effective treatment for posterior elbow impingement and whether it enables athletes to return to a previous level of function.

**Methods:**

A retrospective review of 36 consecutive patients that underwent arthroscopic debridement of the posterior elbow was performed. There were 34 male and 2 female patients, with a median age of 32 years (17–54 years). There were 7 professional athletes, 6 college athletes, and 23 high school or recreational athletes. All patients had a positive posterior impingement test for posterior pain with extension and limitations of activity. Arthroscopic debridement and additional surgical procedures were performed, and patients underwent follow-up visits at a median 51 months (range 14–81).

**Results:**

Significant improvements were seen in pain, motion, and function. No neurovascular complications were seen related to the arthroscopic debridement. The mean Andrews and Timmerman elbow score improved from 159 ± 27 to 193 ± 11 (*p* < 0.01). Thirty-five of thirty-six (97%) patients returned to their previous level of activity, including all professional athletes.

**Conclusions:**

Arthroscopic management of posterior elbow impingement is safe and effective and can return patients, including professional athletes, to high-level athletic activity. Athletes with symptomatic posterior elbow impingement can be successfully and safely treated with arthroscopic debridement and typically will return to preinjury levels of activity.

**Level of evidence:**

IV.

**Electronic supplementary material:**

The online version of this article (doi:10.1007/s00167-017-4563-1) contains supplementary material, which is available to authorized users.

## Introduction

Symptoms of posterior elbow impingement can cause disabling pain and limitation of motion during activities involving elbow extension, including sports activities and manual labour [1, 17, 20, 24, 26, 31–33]. It is a response to repeated high stresses and can be seen in primary degenerative arthritis [36, 39], repeated hyperextension trauma [1, 16, 20], and valgus extension overload syndrome [25, 39]. Posterior impingement of the elbow among professional athletes such as pitchers is not uncommon. The forces generated during the acceleration and follow-through phases of pitching can cause osteochondral changes on the posteromedial olecranon and fossa [9, 21, 24, 29]. In addition, an increased load may be placed on the posterior olecranon due to gradual overload of the ulnar collateral ligament (UCL) [2, 38]. Unfortunately, these injuries can be career threatening. Even among recreational athletes, impingement can cause limitations of athletic enjoyment and activities of daily living. Many of these patients can be treated conservatively with activity modification and anti-inflammatory medication. However, a percentage of impingement patients may need surgical intervention. Surgical intervention is indicated when patients have disabling pain or loss of function despite an appropriate period of nonsurgical intervention, typically 3–6 months. Many patients are only symptomatic during full extension activity such as throwing. Open surgery has been used for the removal of posterior olecranon osteophytes and joint debridement [3, 36, 39]. Arthroscopic debridement of the elbow is a less invasive surgical option [4, 7, 15], with variable results [10, 13, 26, 27, 30–32]. This study attempts to quantify the results of arthroscopic debridement of the elbow as a treatment for posterior impingement in the athletic population and assess its viability for recovery of loss of function. Specifically, it determines whether arthroscopic debridement is a safe and effective treatment for posterior elbow impingement, and enables athletes to return to their previous level of function. This study hypothesizes that arthroscopic debridement can result in effective management of pain and return of range of motion, enabling athletes to return to their previous level of function with minimal complications.

## Materials and methods

**Fig. 1:**
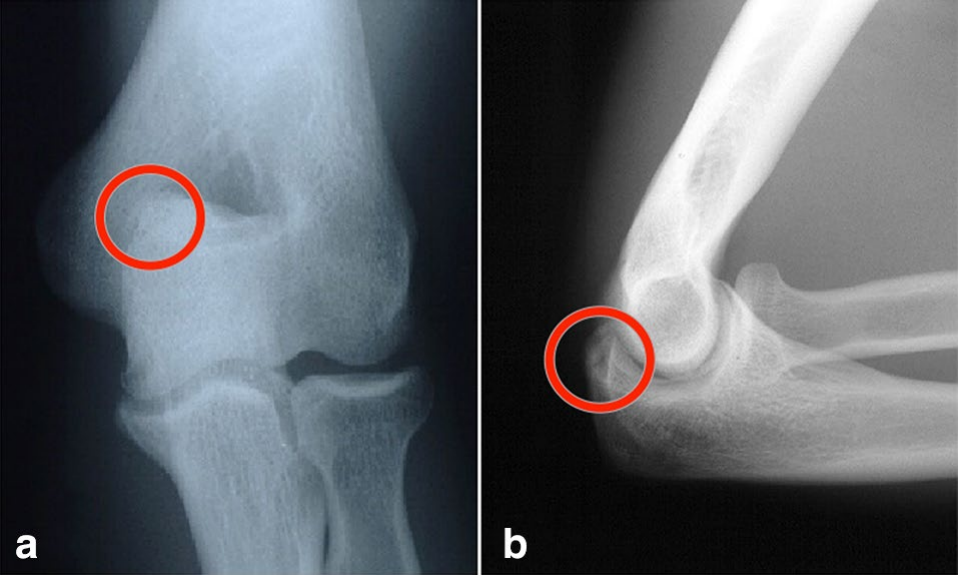
Preoperative radiographs with a circle demonstrating the olecranon osteophyte. **a** The preoperative AP radiograph showing posteromedial olecranon osteophytes. **b** The preoperative lateral radiograph showing posterior olecranon osteophytes

This case series retrospectively reviewed 49 consecutive patients identified as having posterior impingement that underwent arthroscopic debridement of the posterior elbow over a 7-year period. Only patients with pain with functional activity, failure of nonsurgical management, and/or loss of range of motion were included. Patients with significant arthritis, non-athletes, or those who did not experience significant impact due to activities were excluded. Thirteen of the 49 patients had concomitant ulnar collateral ligament (UCL) tears. The following analysis was performed both with and without the UCL tear patients included. Presented here is the data for isolated posterior impingement arthroscopic debridement (UCL tear patients excluded). Of the 36 patients in this cohort, 34 were male and 2 were female patients, with a mean age of 32 years (range 17–54 years). Seven professional athletes, 6 college athletes, and 23 high school or recreational athletes were included in this case series. Athletes participated in the following sports: baseball (*n* = 15), bowling (*n* = 1), football (*n* = 1), golf (*n* = 1), gymnastics (*n* = 1), karate (*n* = 1), mountain biking (*n* = 1), rock climbing (*n* = 1), rugby (*n* = 1), softball (*n* = 2), squash (*n* = 1), swimming (*n* = 1), tennis (*n* = 5), and weightlifting (*n* = 4). Follow-up was available for a median of 51 months (range 14–81). Patients were graded according to the Andrews and Timmerman score [35]. All patients had a diagnosis of posterior elbow impingement by clinical exam and history and had failed conservative management. Patients were examined preoperatively by the senior author and assessed for mechanical symptoms, pain, motion, and level of function. All these patients were not able to participate in their sport at their desired level. Patients would describe posterior pain on terminal extension that could be reproduced by provocative testing (the posterior impingement or “clunk” test) [23, 37]. History of posterior elbow pain between the acceleration phase and follow-through phase of pitching was also felt to be significant [1]. Four patients complained of locking or catching symptoms. Twenty-two patients (61%) experienced a loss of extension averaging 13° (range 0°–30°) measured using a goniometer in office. The goniometer measurement technique was performed similarly to Blonna et al. [8] with an ICC of 0.97 and negligible systematic error of 1°. All patients suffered loss of function (20 limited sports activity, 9 limited other activities, 7 affected activities of daily living). Each of the patients had pain. The mean [26] elbow score was 159 ± 27 with a mean subjective score of 71 ± 15 (pain, swelling, locking, activity) and a mean objective score of 88 ± 18 (contractures, rotation, sagittal arc). AP and lateral radiographs were taken of all patients (Fig. 1a, b). Axial or stress views were not routinely acquired. Posterior olecranon osteophytes were noted in 19 patients (Fig. 1b). Loose bodies were seen in 15 patients. MRI of the elbow was conducted in 13 patients and demonstrated posterior osteophytes in 10. MRIs were not ordered for all patients because radiograph only was felt to be sufficient in some patients. Some patients did not have radiographic evidence of soft tissue osteophytes but had soft tissue contractures or impingement, also causing loss of extension.

**Fig. 2:**
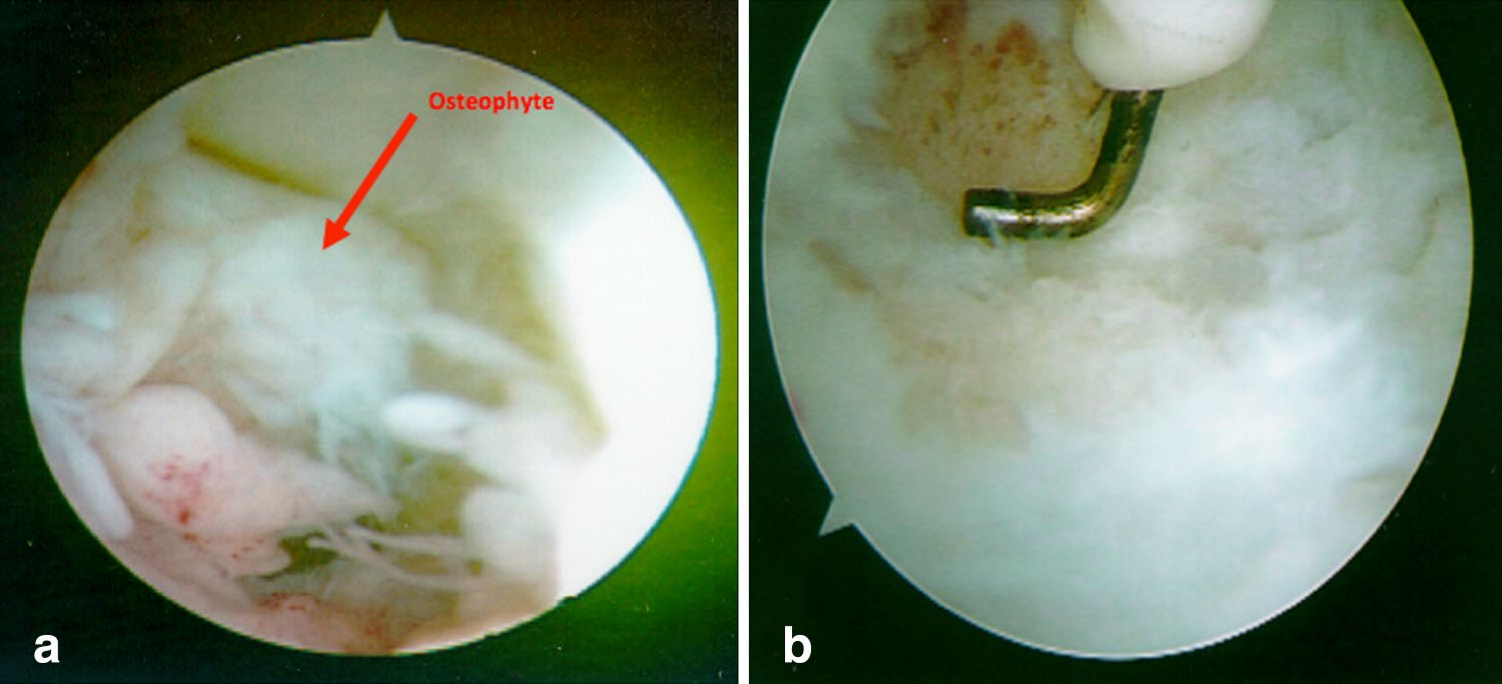
Photographs show arthroscopic views of **a** a posterior olecranon osteophytes with the osteophyte labelled by an *arrow* **b** posterior osteophyte after debridement

The patient was typically placed in the prone position with the arm abducted 90°, and the elbow flexed 90°. If an open ulnar ligament reconstruction or ulnar nerve transposition was planned, the patient was placed in a sloppy lateral position with the arm suspended using an arm holder (McConnell, TX). The joint capsule was distended with 20–40 ml of saline. Diagnostic arthroscopy of the anterior and posterior elbow was performed (Fig. 2a). An electrocautery and 4.5 mm full radius shaver were used to debride the posterior olecranon and olecranon fossa through the posterolateral, and central posterior portals until no posterior impingement was noted on extension (Fig. 2b). The olecranon fossa was not fenestrated. Additional debridement of loose bodies [22], synovium [12, 14], and capsular tissue was conducted as needed. Arthroscopic valgus stress testing was performed before and after debridement to evaluate the UCL [18, 25, 28]. Procedures included osteophyte debridement conducted in 30 patients. Anterior–posterior label radiographs sometimes did not show the olecranon tip osteophytes in all patients, so 10 osteophytes were not identified via radiograph. Due to this, oblique views are recommended to better identify osteophytes. In 2 patients, only soft tissue debridement was performed. Fifteen patients had removal of loose bodies. Eighteen patients had synovial debridement. Four patients had release of scar tissue and capsule. Two patients underwent debridement and picking of osteochondritis dissecans of the capitellum.

Follow-up was available for a mean of 44 months (range 14–81). The surgeon performed the follow-up for all patients. Typically clinical evaluation only was performed. This study recognizes that follow-up for some patients was shorter than 24 months, but their immediate condition was addressed and patients returned to previous level of activity. Follow-up was no longer seen as necessary after patients returned to previous level of activity and was ceased early for these patients only. At follow-up, patients were graded according to the Andrews and Timmerman score [6, 35].

The Institutional Review Board of the Hospital for Special Surgery, New York, provided approval for this study. The IRB approval ID is 2015-639.

### Statistical analysis

Statistical testing was conducted with the use of paired two-tailed *T* test with correction for multiple comparisons. A prospective sample size calculation was not conducted for this study due to the nature of it being a retrospective cohort. A post hoc analysis indicates that for a power of 0.8 and alpha error of 5%, the needed sample size would be 19. The study cohort exceeded this.

## Results

**Fig. 3:**
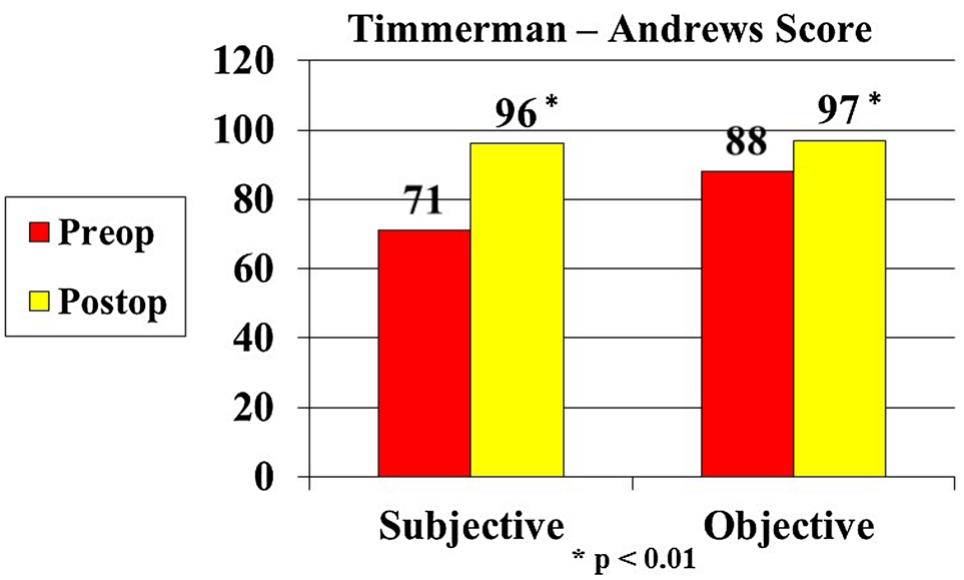
Graph shows Timmerman and Andrews subjective and objective score outcomes

There were statistically significant improvements (*p* < 0.01) in pain, motion, and function, and no neurovascular complications directly related to the arthroscopic debridement. The mean Andrews and Timmerman elbow score improved from 159 ± 27 preop to 193 ± 11 post-op (*p* < 0.01), with the mean objective score improving from 88 ± 18 to 97 ± 7 (*p* < 0.01) and the objective score improving from 71 ± 15 to 96 ± 7 (*p* < 0.01) (Fig. 3). Thirty-four patients (94%) improved in pain. Twenty-three patients had no pain. Twelve patients had occasional pain. Twenty-one out of twenty-two (95%) with limited extension of the elbow improved their range of motion post-operatively. The average improvement for this group was 9° ± 6°. All 4 patients with locking and catching had complete relief of symptoms.

Thirty-five of thirty-six (97%) patients were able to return to desired level of activity. Seven were able to return to professional sports (100%). Five of six (88%) were able to return to college sports. Twenty-three patients were able to return to high school/recreational activities (100%). Return to play was reported by patients as return to previous level of preinjury activity. No transient paresthesias, nerve block complications, prolonged stiffness, or wound complications were noted.

As stated previously, thirteen patients had concomitant ulnar collateral ligament (UCL) tears. When these patients were included in the analysed cohort, the results were similar to the cohort analysed above. With the UCL tear patients included, significant improvements in subjective and objective scores were still observed; however, return to full sports activity was less predictable in this group (three of thirteen were unable to return to desired level of activity). Of the UCL tear patients, eight did not have medial elbow pain with valgus stress at the time of the examination and so UCL reconstruction was not indicated. Five of the eight patients had later UCL reconstruction. Two of these were for new tears; the other 3 had failed conservative management of known ligament laxity. One patient with a concurrent UCL reconstruction developed a medial antebrachial neuroma from the incision for the reconstruction. This was successfully treated with excision of the neuroma. One patient had recurrent effusions in the elbow joint. The histological exam of the synovium revealed inflammatory arthritis and has been treated with steroids and anti-inflammatory medications, but recurrent effusions remained. One patient developed superficial portal cellulitis without evidence of joint infection and was successfully treated with oral antibiotics.

## Discussion

The most important finding of this study was that the mean Andrews and Timmerman score improved significantly for these patients (mean 159 ± 27 to 193 ± 11, *p* < 0.01). Patients in this study had resolution of the pain and loss of function related to posterior impingement after arthroscopic treatment. Arthroscopic treatment was an effective and safe procedure for treatment of posterior impingement. However, this study discovered that a significant minority of the patients (13/49) expressed concurrent UCL laxity and resection of the stabilizing osteophyte may make medial sided pain more evident in these cases. It had been previously established that many activities of daily living can be conducted at minimum within a 30°–130° arc [5, 16, 19, 34]. However, posterior impingement can cause pain and limitations of function in athletes [10, 26, 29, 31] and also can cause pain in certain everyday activities, such as lifting heavy objects [36]. Valgus torque and rapid extension place a force on the elbow. This reoccurring motion can damage the integrity of the UCL [29]. It can also cause posterior impingement due to the forcible extension of the tip of the olecranon into the posterior fossa. Our primary objective was the relief of pain, rather than improvement of motion, similar to the goals articulated in the ulnohumeral arthroplasty work by Tsuge and Mizuseki [36]. In this study patient population, posterior elbow impingement was causing pain and limitation of activity that was not successfully treated non-operatively. This study found that arthroscopic management of posterior elbow impingement is safe and effective in an athletic population.

Posterior elbow impingement is a pathologic process that can occur as part of a spectrum of disease, from soft tissue and synovial irritation in athletes sustaining hyperextension or high valgus extension stress, to degenerative osteoarthritis in heavy manual labourers [36]. Most patients in this study were young, high demand athletes. Few (7/49) had frank osteoarthritis. In this study, patients with primarily degraded systems were excluded. In this study patient population, the olecranon fossa was not fenestrated. The population is different than described by Olgivie-Harris and Schemitsch [27] or O’Driscoll [22]; these groups had significant losses of motion and degenerative disease. More comparable groups would be those treated by Andrews and Timmerman [6] and Ward and Anderson [37], a younger, more athletic population.

In these athletes, it is important to suspect and examine for valgus instability, particularly in throwers. The patients in this study had a high incidence of concurrent injury, consistent with the valgus extension overload mechanism. Specifically, 11/49 (22%) had known or diagnosed UCL tears at time of surgery. Many of these MRI diagnosed patients did not have medial elbow symptoms and declined UCL reconstruction. However, half of these failed conservative treatment and required UCL reconstruction. In addition, 2 patients tore their UCL during the follow-up period. A total of 13/49 (27%) had associated tears. Five of these patients had simultaneous reconstructions while the others underwent previous reconstructions. This was consistent with Andrews and Timmerman’s study of professional baseball players (25%) [6]. It is critical to note that the medial pain at roughly 60°–100° of elbow flexion associated with UCL insufficiency was distinct from the posterior pain at the olecranon tip at full extension seen in posterior impingement. This study found the arthroscopic valgus instability test to be a valuable tool to assess the UCL [18]. Several abstracts have presented a relatively high rate of failure with posterior decompression. It is unclear whether there may have been unrecognized laxity contributing to the high re-operation rate.

Complications were few in this population. Elbow arthroscopy is a relatively high-risk procedure [6, 11], and neurovascular structures are quite close to the joint. The hypertrophic bone in overhead throwing athletes is typically posteromedial, close to the ulnar nerve. Surgeons in this study were cautious in working in this region, using limited electrocautery with good saline flow, and very limited suction of the shaver. This study experienced no patients with ulnar nerve complications.

Arthroscopic treatment of posterior impingement has several advantages when compared to open surgery. A few early reports indicated that its usefulness was limited [27, 32]; however, more recent reports concur in its utility [10, 24, 26, 31, 33]. A limited decompression can be performed without disruption of the extensor mechanism or collateral ligaments and preserves the normal bony stability of the joint. Arthroscopy allows the careful inspection of the entire joint, including assessment of the UCL. Concurrent procedures, such as synovial debridement [12, 14] and removal of loose bodies [22] can also be performed. Arthroscopic surgery also does not limit other, open procedures from being performed.

Despite the overall success of treatment, some patients did not improve to a level that they were able to return to a previous level of activity. A few of these patients had relatively extensive degenerative disease, which the surgeons were unable to completely treat. Other patients were not able to progress in their high school or college careers as they had wished; this may have been related to the time needed to rehabilitate after combined arthroscopic debridement and open ligament reconstruction. However, the majority of patients and all professional level players were able to return to their previous level of play. If continued repetitive overload stress occurs, there is a possibility of recurrence of posterior impingement in these athletes over time. However, in this population the results of the arthroscopic debridement procedure were clinically durable, and none of the patients over the course of treatment required repeat debridement. The authors of this study have found that radiographs taken with the arm in elbow flexion and externally rotated on a plate can help provide a clear view of the posterior olecranon tip.

This study had a number of limitations. First, as a study population, the group was mixed with several patients having a posttraumatic or other diagnosis. However, the majority of the patients had mild degenerative disease related to sports or work activity, and all patients had characteristic findings of posterior pain with extension. To that extent, the clinical presentation was very homogenous. Second, this study was a retrospective series with a variety of athletic injuries. However, the straightforward nature of the clinical question allowed the use of a retrospective review and that all major and minor complications were accurately recorded. In addition, a percentage of these patients had concomitant findings. However, it was felt important to note that in many cases this problem does not occur in isolation. The positive outcomes of arthroscopic debridement treatment of posterior elbow impingement are encouraging. These results further support arthroscopic debridement as the recommended treatment of posterior elbow impingement as it is less invasive than many of the alternative approaches.

## Conclusions

In this patient population, 94% of patients experienced a decrease in pain, and 97% were able to return to their desired level of activity. It was found that arthroscopic debridement of the elbow, in the hands of experienced elbow arthroscopy surgeons, is a safe and effective treatment modality to relieve pain, improve function, and assist in returning athletes to their previous level of function.

## Electronic supplementary material

Below is the link to the electronic supplementary material.

Supplementary material 1 (DOC 27 kb)
