# Supplementary material for: Arthroscopic treatment successfully treats posterior elbow impingement in an athletic population
Source: Knee Surg Sports Traumatol Arthrosc. 2017 May 22;26(1):306–11. doi: 10.1007/s00167-017-4563-1 (PMC5754399; doi:10.1007/s00167-017-4563-1)
Supplement: Supplementary file 1 — Supplementary material 1 (DOC 27 kb) [file 167_2017_4563_MOESM1_ESM.doc]

Table 1 Scoring system (from Timmerman and Andrews [35])

Scoring Subjective/objective Overall

Excellent 90-100 180-200

Good 80-89 160-179

Fair 60-79 120-159

Poor <60 <120

Subjective Points

## Pain

None 25

Occasional 20

With moderate activity 10

With ADLs 5

## Swelling

None 25

Occasional w/heavy activity 20

With moderate activity 10

With any activity 5

## Locking/catching

None 25

Rare 20

Occasional 10

Frequent 5

## Activities

No limit 25

Occasional limit 20

Partial activities only 10

Unable to work/ 5

difficulty with ADLs

Objective Points

## Flexion contracture

<5^o^ 25

5 ^o^ -15 ^o^ 20

16 ^o^ -35 ^o^ 10

>35 ^o^ 0

## Pronation/supination

Normal 25

<30% decrease normal arc 20

<50% decrease normal arc 10

>50% decrease normal arc 0

## Sagittal arc of motion

>130 ^o^ 50

120 ^o^ -130 ^o^ 40

110 ^o^ -119 ^o^ 30

100 ^o^ -109 ^o^ 20

75 ^o^ -99 ^o^ 10

60 ^o^ -74 ^o^ 5

<60 ^o^ 0
